# The geography of references in elite articles: Which countries contribute to the archives of knowledge?

**DOI:** 10.1371/journal.pone.0194805

**Published:** 2018-03-26

**Authors:** Lutz Bornmann, Caroline Wagner, Loet Leydesdorff

**Affiliations:** 1 Division for Science and Innovation Studies, Administrative Headquarters of the Max Planck Society, Munich, Germany; 2 John Glenn College of Public Affairs, The Ohio State University, Columbus, Ohio, United States of America; 3 Amsterdam School of Communication Research (ASCoR), University of Amsterdam, Amsterdam, The Netherlands; KU Leuven, BELGIUM

## Abstract

This study asks the question on which national “shoulders” the world’s top-level research stands. Traditionally, the number of citations to national papers has been the evaluative measures of national scientific standings. We raise a different question: instead of analyzing the citations to a countries’ articles (the forward view), we examine references to prior publications from specific countries cited in the most elite publications (the backward—citing—view). “Elite publications” are operationalized as the top-1% most-highly cited articles. Using the articles published from 2004 to 2013, we examine the research referenced in these works. Our results confirm the well-known fact that China has emerged to become a major player in science. However, China still belongs to the low contributors when countries are ranked as contributors to the cited references in top-1% articles. Using this perspective, the results do not support a decreasing trend for the USA; in fact, the USA exceeds expectations (compared to its publication share) in terms of references in the top-1% articles. Switzerland, Sweden, and the Netherlands also appear at the top of the list. However, the results for Germany are lower than statistically expected.

## 1 Introduction

The invention of the *Science Citation Index* in the 1960s was welcomed by citation analysts as well as historians and philosophers of science as offering an opportunity to study science empirically [1, 2, 3, 4, 5]. As it developed, citation analysis split into two research traditions with different perspectives on the reference: citation analysts are interested in counting “times cited” as a measure of quality or impact to aid evaluation; references can be used in the history and philosophy of science as a tool to retrieve “revolutions and reconstructions” in science [6]. Wouters [7] noted that transposing the cited/citing matrix by the database owner adds value: the references become citations. The *Science Citation Index* thus shaped the field of citation analysis as a domain analytically different from historical reconstructions.

The two perspectives are reflected in citation analysis as the difference between co-citation [8, 9] and bibliographic coupling [10]. By citing, a scholar reconstructs the intellectual context of one’s knowledge claim [11]. Being cited, however, is rewarding in terms of providing credit and reputation [12]. The intellectual organization of the sciences, however, evolves in—often anonymized—texts that are cleaned in a process of validation from the contingencies of the context of discovery [13]. In this study, we focus on the relative positions of countries: what is referenced in the worldwide top-level research in terms of national contributions?

Which countries provide the longer-term intellectual context of top-level research? We are inspired to assume this approach by bibliometric analyses for the Science and Engineering Indicators report of the US National Science Foundation [14]. In this context, Mervis [15] covered the shares gained by specific countries (and transnational units such as the European Union) in the 1% most-highly-cited papers. These shares were size-normalized by using the countries’ numbers of published papers as a baseline. In order to reveal on which “shoulders” the worldwide top-level research stands, we decided upon a similar approach as Mervis [15], but used the cited references instead of the citations perspective [16, 17]: In other words, we investigated which country’s literature is incorporated in the archive of work cited at the top of the pyramid.

Operationally, we include the 21 countries in this study that published more than 1% of the articles worldwide during the period under study. Using the years 2004 to 2013, we investigate a substantially longer and more recent time period than Mervis [15]. We focus on countries as units of analysis for three reasons: 1) national systems represent underlying cultural, social, economic, and political models; 2) national governments seek to encourage knowledge creation, diffusion, and exploitation; and 3) most basic research is paid for by public funds. In addition to activities at the research front, participation, prestige, and the build-up of intellectual capital are longer-term objectives of national policies. Prestige can be considered as generalized from performance [18].

Reputation and prestige have real consequences for attracting resources and market shares. For example, the label “Made in Italy” has a value that can be compared with “Made in China” in terms of assumption about quality; the ‘capital’ or reputation attached to “Made in Italy” has been built up over time and with attention to maintaining quality. Prestige in science attracts foreign students and collaborators who can contribute to the vitality of a system [19, 20, 21, 22, 14]. For this study, we consider publications as investments in intellectual capital, reflecting the investments made in maintaining quality. Citations of publications can be a way of measuring a return on investment [23]. To what extent do authors of top-1% publications make references to contributions from the same country or internationally?

In other work, we showed that, in terms of field-normalized performance for the top-1% and top-10% most-frequently cited publications, the USA held the lead in the 2000s, but increasing numbers of citations were going to EU28 nations, which had also increased their share of articles in the top 10% highly cited articles [24]. Several smaller European nations—Switzerland, Denmark, Sweden, and the Netherlands—had surpassed the USA in percentage share of highly cited articles. As noted, Mervis [15] showed that Asian scientists increasingly cite other Asian articles. Mervis’ [15] report was limited to only five countries or aggregates of countries (e.g., the Asia-8 and the EU). In this study, we expand upon Mervis’ [15] report and use the cited references to view national contributions to the archives of knowledge for the 21 countries that contributed 1% or more of all published material with the document type “article” in Web of Science (WoS) between 2004 and 2013.

## 2 Data and methods

The 21 countries that contributed 1% or more of all published material with the document type “article” in Web of Science (WoS) between 2004 and 2013 published 86% of all articles indexed in these years (across all subject categories). Using this 1% threshold, most countries worldwide with a substantial contribution to the archive are included; however, small-sized but potentially top-performing countries in terms of relative citation impact such as Denmark are unfortunately excluded because of the threshold [24].

From the set of all articles published between 2004 and 2013, we select the top-1% most highly cited research worldwide. We call these papers “elite” articles. “Elite” articles are those articles which belong to the 1% most-frequently cited papers in the corresponding WoS subject categories and publication years. From this set of articles, we use all the cited references. This results in a subset of the data that we further cleaned in three ways, removing about 40% of the material. 1) We include only references to papers with the document type “article”. 2) We remove articles lacking country information in the address lines—otherwise we could not link knowledge contributions to countries, which is the point of the analysis. 3) We eliminate referenced articles dated prior to 1980 because the database does not contain reliable address information prior to this date.

The final data contains articles listing one or more countries in the address lines. If an article has multiple country names in the address lines, we count contributions to these articles fractionally based upon the numbers of countries listed. The count goes to countries—not to authors: if multiple authors are listed from the same country, the count is still “one” for this country. If two authors from two different countries are listed on the article, the article is assigned to each country with a value of 0.5; for three countries, the value is .33, and so on.

Table 1 shows the countries with the largest shares of all articles between 2004 and 2013. As expected, the United States (USA) is at the top of the list of countries, followed by China, Japan, the UK, and Germany as the countries contributing the largest numbers of articles. (The European Union is not considered as a single unit in this analysis.) China appears second in the total numbers of articles. However, it did not begin the decade in this second position, but grew much more rapidly than other countries to finally claim the second spot [25].

**Table 1:** Twenty-one countries with the largest shares of all articles indexed in WoS between 2004 and 2013. Only countries with more than 1% of the fractionally-counted articles are listed, in decreasing order of the percentage share of articles.

Country	Absolute numbers of articles (full counting)	Absolute numbers of articles (fractional counting)	Percentages of share (fractionally counted)
USA	3,168,104	2,634,682.58	24.02
China	1,235,872	1,080,633.73	9.85
Japan	749,737	642,650.14	5.86
UK	852,450	610,480.36	5.57
Germany	825,301	588,873.30	5.37
France	591,754	415,985.78	3.79
Canada	499,266	368,465.44	3.36
Italy	469,169	348,992.92	3.18
India	367,526	324,676.10	2.96
South Korea	364,148	309,488.83	2.82
Spain	403,738	302,138.84	2.75
Australia	354,499	260,940.89	2.38
Brazil	276,151	233,401.27	2.13
Russian Federation	264,695	212,192.69	1.93
Taiwan	219,447	192,525.35	1.76
Netherlands	276,224	186,951.06	1.70
Turkey	197,794	178,076.95	1.62
Poland	178,423	140,765.21	1.28
Sweden	189,527	126,237.57	1.15
Switzerland	201,586	122,881.07	1.12
Iran	137,972	122,174.78	1.11

## 3 Exploring international contributions

Table 2 lists countries in the same order as Table 1, but it shows the number of references per country and their respective shares of contributions to elite publications. For example, China published 9.85% of the worldwide articles (Table 1), but it contributed only 4.24% of cited references in the top-level research papers. The opposite is seen for the USA: it contributes 24.02% of worldwide articles (Table 1) and 44.1% of the references in top-1% (Table 2). This large share for the USA led us to consider weighting the data, since a factor accounting for the differences may be the publication volume of the various countries [26]. The more articles a country has published, the more citations can *ceteris paribus* be expected.

**Table 2:** Country counts from cited references in elite publications. Sorting order matches Table 1.

Country	Absolute numbers (full counting)	Absolute numbers (fractional counting)	Percentage share (fractionally counted)
USA	1,690,279	1,403,550.91	44.10
China	177,660	134,969.53	4.24
Japan	194,537	150,026.31	4.71
UK	378,794	247,783.00	7.79
Germany	299,068	184,287.05	5.79
France	204,445	121,722.31	3.82
Canada	199,270	128,511.17	4.04
Italy	136,224	79,005.36	2.48
India	35,543	26,136.91	0.82
South Korea	53,244	37,210.09	1.17
Spain	91,964	54,105.14	1.70
Australia	122,767	77,797.85	2.44
Brazil	27,888	15,223.68	0.48
Russian Federation	30,163	13,051.16	0.41
Taiwan	30,409	22,615.22	0.71
Netherlands	132,073	78,608.76	2.47
Turkey	16,041	12,018.90	0.38
Poland	24,378	11,146.73	0.35
Sweden	81,830	47,488.45	1.49
Switzerland	102,371	56,292.08	1.77
Iran	8,092	6,366.80	0.20

**Fig 1:**
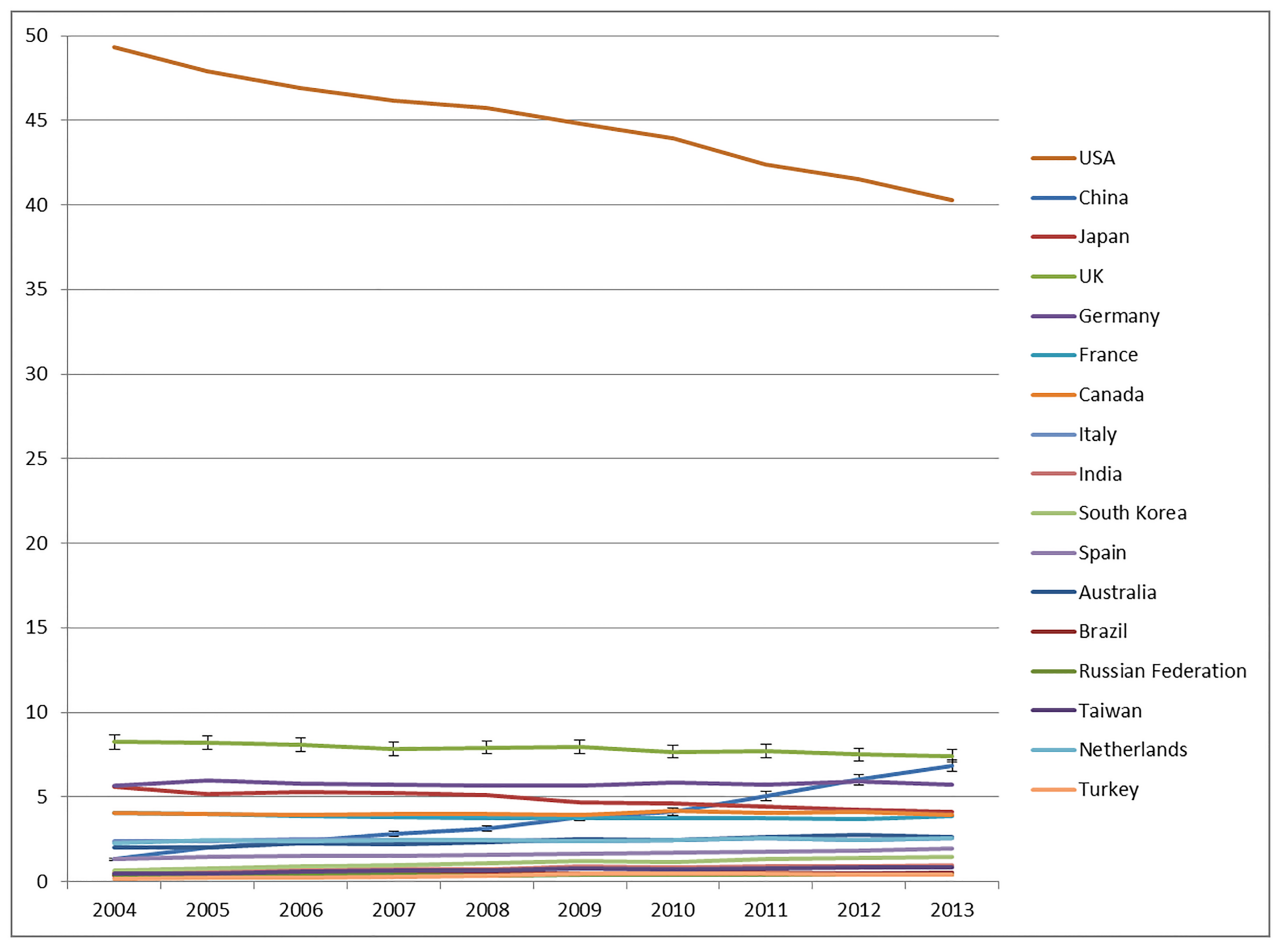
Countries’ shares of references cited in the elite articles between 2004 and 2013 (articles belonging to the 1% most frequently cited articles, fractionally counted). 95% confidence intervals are added to the UK’s and China’s shares.

Fig 1 shows the same data as Table 2, but broken down by year of publication. As expected, the USA’s contribution is decreasing [27], whereas China’s contribution is increasing over the years. The USA’s share of cited references in top-1% articles dropped by approximately nine percentage points—more than the drop in publication volume [24]. However, China’s share increased by 5.7 percentage points, which is proportional to its gain in shares of publications. We included 95% confidence intervals in Fig 1 for China and the UK as interval estimates indicating the accuracy of our point estimates (the percentages) [28]. The absence of overlap of the 95% confidence intervals for the UK and China shows that the lead of the UK versus China is (still) statistically significant at the end of the period [29, 30]. The shares of the other countries are more or less constant over the years.

In Table 3, we report the ratio of cited references in the elite articles to citing articles for each country. Assuming that many papers reach their citation peak in the third year after publication, we use the ratio of cited references in year *t* and published articles from year *t*-3. This ratio reveals whether a country received more citations than expected on the basis of the number of published articles. The findings show that the USA has an average ratio of 1.7 (cited references) and (citing articles) during this period. Thus, the USA contributed much more to the archive of knowledge than can be expected on the basis of its publication volume. Maisonobe, Milard, Jégou, Eckert, and Grossetti [31] report on similar results for the US. Besides the US, Switzerland, the Netherlands, the UK, and Sweden had higher-than-expected citedness compared to publication volume in this study.

**Table 3:** Mean ratios and standard deviations of shares (cited references versus citing articles) across 10 years as well as the difference between the ratios in the last (2013/2010) and first years (2004/2001) (sorted by the means).

Country	Mean	Rank order	Standard deviation	Rank order	Last year—first year	Rank order
USA	1.71	1	0.03	17	0.03	17
Switzerland	1.53	2	0.08	5	0.21	6
Netherlands	1.39	3	0.09	3	0.28	2
UK	1.29	4	0.08	6	0.21	5
Sweden	1.20	5	0.06	8	0.12	11
Canada	1.17	6	0.03	16	-0.02	20
Australia	1.04	7	0.09	4	0.18	7
Germany	0.99	8	0.10	2	0.27	3
France	0.91	9	0.07	7	0.22	4
Italy	0.75	10	0.04	12	0.12	10
Japan	0.69	11	0.05	9	0.11	12
Spain	0.62	12	0.04	11	0.16	8
China	0.50	13	0.10	1	0.34	1
South Korea	0.45	14	0.04	13	0.12	9
Taiwan	0.42	15	0.04	14	0.10	13
India	0.30	16	0.03	19	0.03	16
Poland	0.28	17	0.02	21	0.01	18
Iran	0.27	18	0.05	10	0.07	14
Brazil	0.26	19	0.03	18	-0.03	21
Turkey	0.25	20	0.03	15	-0.01	19
Russian Federation	0.19	21	0.02	20	0.07	15

Note. The rank positions were calculated on the base of mean values, which are not rounded to two decimal places.

Table 3 shows the mean ratio and standard deviation of shares (cited references versus published articles) that were calculated across 10 years, as well as the difference between the ratios in the last (2013/2010) and first years (2004/2001). By using the mean values, the countries can be categorized into three groups indicated as grey-shaded areas in the table: high, average, and low performers. The high performers have a ratio of at least 1.2, which means that they received substantially more citations in the elite publications than would be expected by publication volume (on average across the years). The average performers approximately meet the expectations (values between 0.8 and 1.19). The low performers fall significantly below the expectations (below 0.8) based upon volume. Table 3 shows that China is still on a low performance level (rank position for the mean is 13), but grows more quickly than the other countries across the years (rank position for the standard deviation is 1). The differences between the last (2013/2010) and first years (2004/2001) in the table point out that China has a high increase in its ratio (0.34), but other countries have a positive showing as well (e.g., the Netherlands = 0.28 and Germany = 0.27).

**Fig 2:**
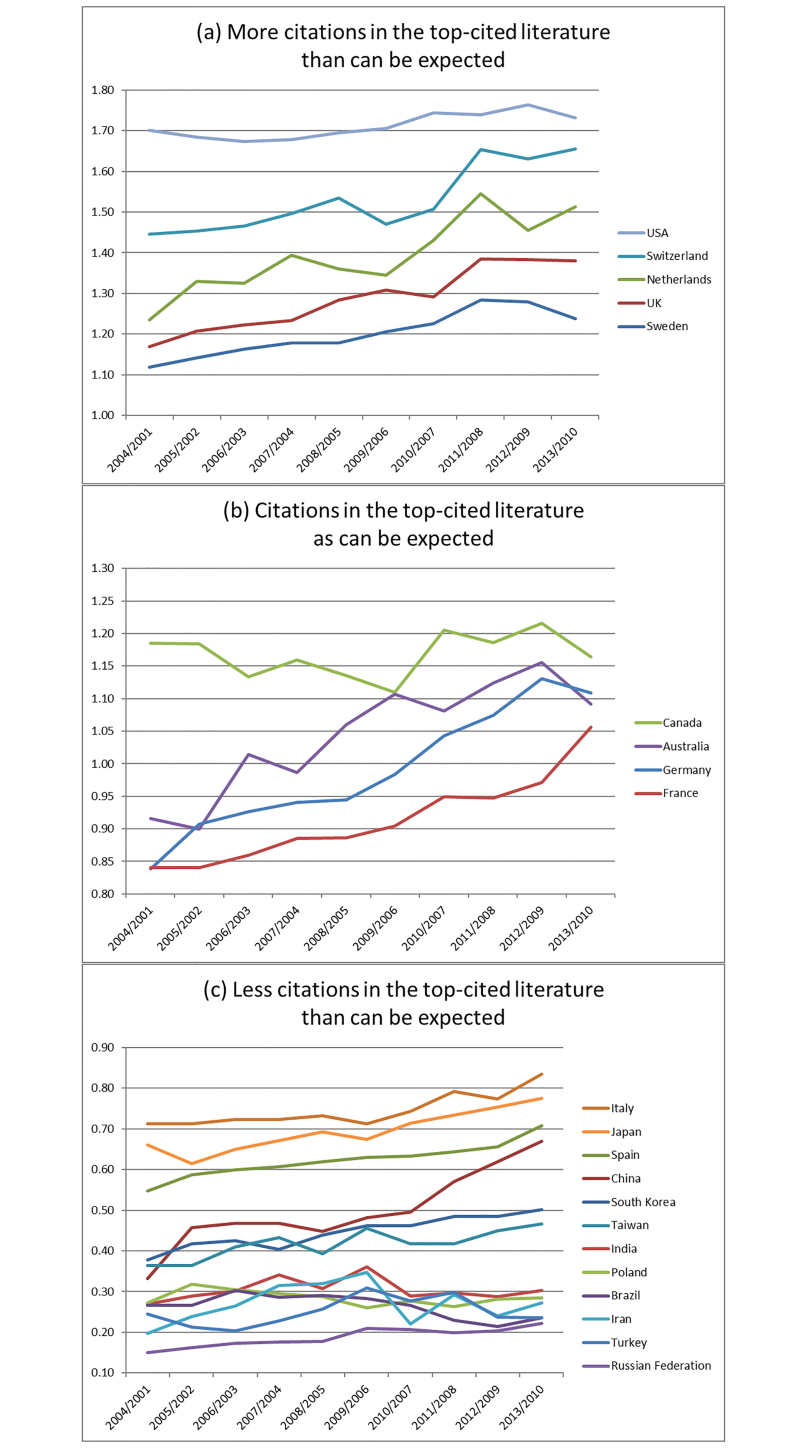
Ratios of shares based on (1) cited articles: countries’ shares of references cited in the top-level research (fractionally counted). (2) Published articles: countries’ shares of articles published between 2001 and 2010 (fractionally counted). The countries are categorized as high (a), average (b), and low (c) performers (see Table 3).

Fig 2 shows the developments of the countries’ ratios of cited references versus citing articles over time. The countries are categorized into three groups of performers as per Table 3: the top box includes USA, UK, the Netherlands, Sweden, and Switzerland—the high performers. Since the group consists of both large and small countries (in terms of published articles), the size-normalization used above seems to function properly. As Fig 2a reveals, the USA performs at the highest level across all years. In recent years, Switzerland has reached a very high level, too. The UK and the Netherlands show an increasing trend.

The average performing group in Fig 2b consists of four countries with ratios around 1 across the years (Germany, France, Canada, and Australia). With the exception of Canada, these countries show an increasing trend. The largest number of countries (n = 12), however, are categorized as belonging to the low-performing group, shown in Fig 2c. Several countries show an upward trend, notably China, which shows an upward turn beginning in 2005. Other countries also show upwards trends, including Italy (since 2009/2006), which performs in the average range by the end of the decade.

## 4 Exploring domestic contributions

As a second research question, we are able to distinguish between each country’s contribution to the archive of the international literature versus the domestic return on investment: how much does a country itself profit from this longer-term incorporation of its contribution to the elite literature? We operationalize this domestic effect by normalizing the country-level contributions to the cited references against the set of elite articles published by the country without considering internationally (co)authored articles. Thus, we focus on the countries stand-alone strength by including only domestic (cited) references and (citing) top-1% articles.

**Fig 3:**
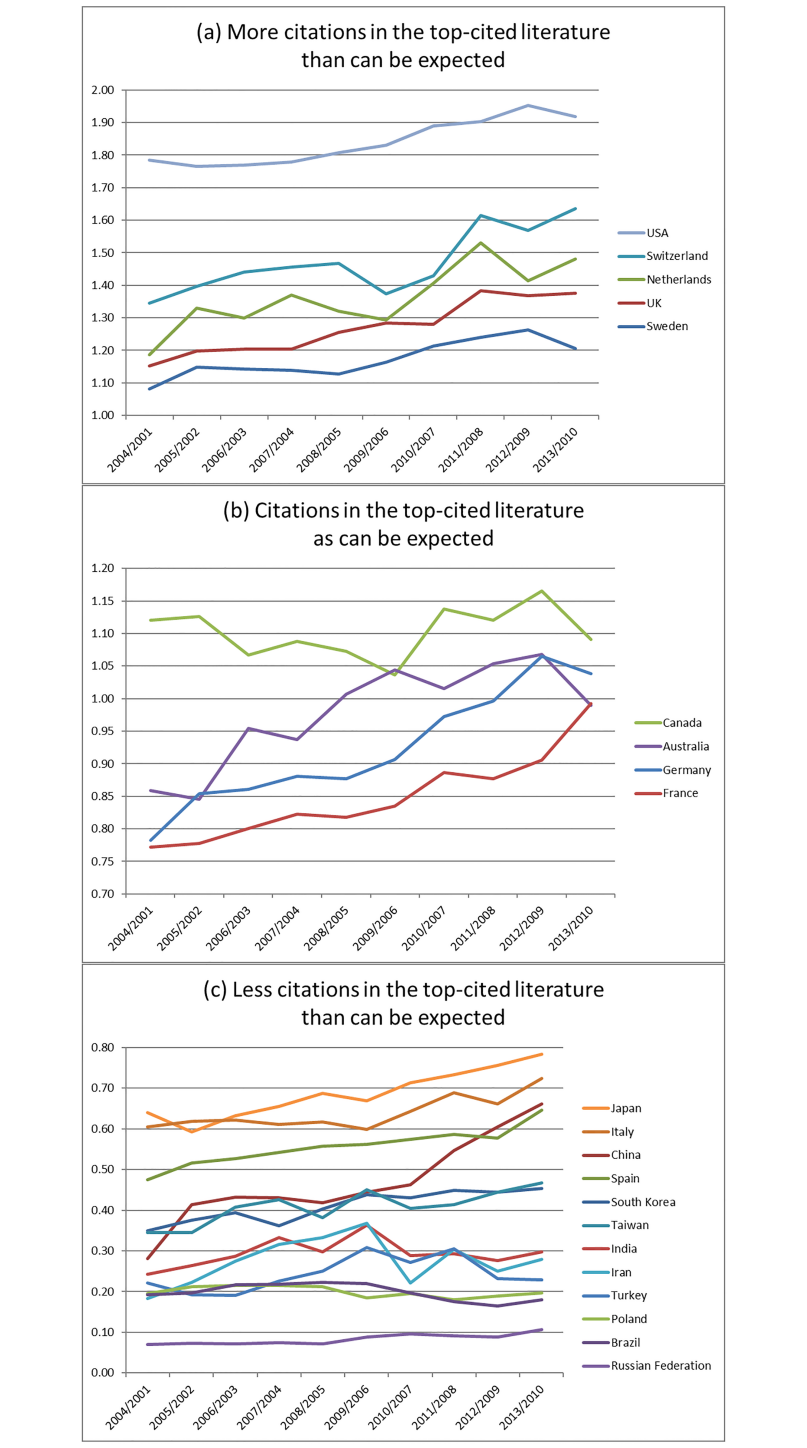
Moving averages of ratios of shares between (1) countries’ shares of references cited in elite articles (fractionally counted) and (2) countries’ shares of articles published between 2001 and 2010 (fractionally counted). The countries are categorized as high (a), average (b), and low (c) performers (as listed in Table 3).

The result is shown in Fig 3, which is rather similar to Fig 2. However, it is the similarity that is telling. The focus on domestic articles in Fig 3 supports the previous results and suggests that the global-level contributions reflect the national efforts and strengths. However, two interesting differences are visible for countries in the top group: 1) When the analysis is limited to domestic articles, the excellent performance of the USA becomes *more* pronounced, suggesting that US authors of papers in the top-1% articles are more likely to cite other US work than work from abroad. 2) Since there is a larger gap between the USA and the other countries, the contribution of the USA seems to reflect its domestic strength.

We suggest that the more articles from a country receive citations in its own top-level research papers, the more it can be considered to have contributed to its own knowledge base. Researchers in a country alternatively can cite international literature more than domestic papers or one can find an overrepresentation of domestic literature, reflecting self-reliance at the national level [32]. With a high share of cited references in the national top-level research (as shown in Fig 3), the investments in science seem to be used efficiently, at least from the perspective of a national government.

Since the shares of cited references cited in the national top-level research papers is not only dependent on the quality of research, but also on the publication volume, the shares of cited references have to be size normalized. After normalization, the comparison of country shares in cited references and citing articles can reveal how a country’s publication system uses the nation’s investment in research. It can further reveal whether these investments spill-over the national borders. In order to make this assessment, the domestic shares of cited articles from each country are contrasted to the shares of published articles. If the ratio is larger than 1, a benefit to the country can be inferred, or, a “gain” of investment from a national perspective**.**

Table 4 shows the result of this domestic analysis for the five countries identified as top-performers. The ratio includes 1) the share of cited references in a country’s top-level research papers that were published by the country itself among all cited references from that country. 2) The share of articles published by the country among all published articles. For example, the share of domestically cited references among the total of cited references in the top-level research articles from 2005 is 66.71% for the USA. The share of US articles among all articles worldwide in 2002 is 28.47%. The resulting ratio is 2.34.

**Table 4:** Ratios of two shares: (1) share of the number of cited references in the country’s articles and the number of that part of cited references which were published by the country itself. (2) Country’s shares of articles published between 2001 and 2010 (fractionally counted).

Year	USA	Netherlands	UK	Switzerland	Sweden
2004/2001	2.34	7.22	3.60	9.87	9.97
2005/2002	2.34	8.00	3.46	10.10	8.23
2006/2003	2.38	7.76	3.71	10.78	8.97
2007/2004	2.41	8.08	3.64	9.94	9.33
2008/2005	2.48	7.48	3.66	9.98	10.09
2009/2006	2.50	7.60	3.86	8.77	9.24
2010/2007	2.56	6.50	3.34	7.25	8.18
2011/2008	2.59	7.03	3.60	8.63	9.30
2012/2009	2.70	6.63	3.78	7.87	8.36
2013/2010	2.67	7.13	3.66	7.48	7.58

Return on investment at the national level far outperforms expectation for the Netherlands, Sweden, and Switzerland: domestic articles by authors in these countries are significantly more frequently cited in the top-level research than one would expect from their respective shares of published papers. However, the trend for Switzerland is decreasing. Thus, Switzerland’s research becomes less important for its own top-level research papers across the years.

## 5 Discussion

When Garfield [33, 34] introduced the Journal Impact Factor (JIF) as a two-year moving average of journal citations, he based this decision on Martyn and Gilchrist’s [35] evaluation of British scientific journals. However, these authors had focused mainly on journals in molecular biology and biochemistry. In these fields, there is a rapidly moving research front with more than 25% of the citations provided in the first two years after publication [36]. The JIF thus discounts the effects of “citation classics” [37] since it privileges the rapidly moving research fronts.

The sciences differ in terms of how relevant this “research front” is for the development of a field [38]. Short-term citation at a research front can be distinguished from longer term processes of incorporation and codification of knowledge claims into bodies of knowledge. Citation classics may not be highly cited in the first few years, but peak later [39].

For example, the *American Journal of Sociology* (AJS) and *American Sociological Review* (ASR)—two leading sociology journals—have cited and citing half-life times of more than ten years. In other words, more than half of the citations of these journals are from issues published more than ten years ago, and more than half of the references are to publications older than ten years. Coleman’s [40] study entitled “Social Capital in the Creation of Human-Capital,” for example, became a most highly-cited paper almost two decades after its publication [41]. Citation classics are not decaying as the citation curves of “normal science.”

This raises a caution in focusing on short-term impact, because one measures, from this perspective, not quality but variation in the positioning of the contribution at the research front. The selection mechanisms of high quality can be expected to develop much more slowly [6]. By choosing long citation windows and only top-1% citing papers, we focus on the most notable scientific papers that are referenced by the top papers. Our approach is “citing” as different from “cited:” using the top-1% elite papers—normalized for fields of science—we can retrieve co-reference patterns (bibliographic coupling [10]) among previous literature.

In a so-called “linked” citation database, one can retrieve bibliometric characteristics of the referenced literature as backward citation and/or forward citation rates. In this study, we focused on the geographical origins of the knowledge contributions by elaborating on a design similarly reported by Mervis [15]. We used the addresses in the bylines of the “citation classics” to attribute them to countries. In order to avoid noise by imprecise referencing in the margins of scientific developments, we focused on the top-1% elite of scientific papers (normalized for fields). One can assume that these authors of top-level articles have worked very carefully on their papers, including highly precise and selective referencing.

Based on previous studies, we expected to see a wider field of contributing countries to these top-level papers, but our results show that the US science system is very strong in contributions to the global knowledge pool, has a persistent reputation, and is heavily relied upon as the source of knowledge by both US and non-US authors. The USA remains the center of science in terms of the citation practices of other scientists who are seeking to advance research. Although the USA may be losing ground in science in other respects [21, 42, 43], this analysis suggests that American authors contribute more to the archives of elite global science than would be indicated by its number of published articles. The size-normalized contribution reveals that the USA has gained ground over other countries instead of losing it. This becomes especially visible if the analysis focusses on domestic publications.

Rodríguez-Navarro and Narin [44] who compared the European Union with the USA have published similar results. Their analyses of publications belonging to the 1% most frequently cited demonstrate the ongoing dominance of the US in science. On the other side, the analyses of Rodríguez-Navarro and Narin [44] also reveal the deficiencies of the European Union in the top-level segment. The European Union is, however, a very heterogeneous set of countries in terms of scientific performance. The results of our analyses show that Switzerland, Sweden, and the Netherlands have high contributions to the cited references used in the top-1% elite articles, as has the USA—a finding similar to results of studies showing these nations as garnering more citations to their work. The surplus-capacity of these countries, measured by the shares of cited references and citing articles, is very high, putting them as among the most productive (and probably effective) of elite science in the world. These high performing countries exist alongside many other European countries with comparably medium or low performance.

Somewhat to our surprise, our results show that Germany does not belong to the top-performing group of countries. This result differs from the results of impact-oriented studies, which have demonstrated high performance for Germany in recent years [45] and historically [23]. The results also differ from the results which have been recently presented by Abbott [46] in a *Nature* comment. The comment is entitled as “the secret to Germany’s scientific excellence” and the presented numbers (e.g., the field-weighted citation impact) “tell a positive story for science” (p. 22). While German articles generally show strong short-term citation impact, Germany’s long-term contributions to the elite literature is not as strong.

It has been argued that German scientists are not as likely to publish in high impact journals as others [47], which may reduce visibility of German research. It appears that German scientists spend less time on collaboration than peers in other top nations [48] and this may reduce the opportunity to contribute to cutting-edge problems. However, one can also argue that Germany is successful in optimizing profit from its investments by maintaining a national publication system. This question of Germany’s efficiency requires further study.

The analysis further confirms that China has emerged as a major player in science, at least in terms of numbers of articles [49, 50, 25]. We expected to find that others increasingly draw upon China’s science; however, our analysis suggests that China’s contributions to the literature are still less relevant for elite publications. There may be many reasons for this beyond quality of research—social networks and language capabilities play roles in the dissemination of scientific knowledge, and these may remain obstacles for many Chinese scientists [51, 52].

Two other countries deserve mention. This is the poor showing of the Russian Federation and Japan. These two nations have declined in science from former leading positions. King [23] showed Japan as the fourth strongest country in the world using data from 1997 to 2001 based upon the top 1% of highly cited publications. In this study, we show that Japan is contributing to references in elite research articles below expectation. This may be due, in part, to the fact that Japan is among the least internationalized nations in percentage terms [53]. Within Japanese culture, it is important to publish findings in Japanese language journals, which may reduce the dissemination of knowledge.

The position of the Russian Federation is more difficult to interpret because historical continuities have been disrupted by the break-up of the Soviet Union. Even so, in the 1990s, Russia was an average performing country, counted by King [23] as close to Finland and Denmark in producing science and claiming citations. Unlike Japan—which has continued to fund R&D at a high rate—Russian investment in R&D has declined. As in Japan, publishing in the national language is important in Russia for one’s reputation; but this orientation may hinder international visibility.

## 6 Conclusions

The measure presented in this paper is a way to reveal the “shoulders” on which worldwide research stands. Rather than a measure of impact—that is, citedness—this measure examines references in top-papers to see from where top researchers draw for their knowledge base. Thus, it is more a measure of prestige and reputation than research impact. We expect that ensuing credit of top-cited papers is persistent since it does not reflect the frontiers, but the archive. In this article, we show the persistent position held in the archive by the United States, the rise of recognition of Chinese papers, and a stable stronghold for Switzerland, Netherlands, and UK; Russia and Japan have a poor showing.

The available measures allow us to examine the extent to which a country’s authors rely upon national work as opposed to foreign research. This can be considered a measure of return on national investment in the research base. The Netherlands, Sweden, and Switzerland far exceeded expectations in terms of using national research in their work, perhaps indicating a strong return on investment. However, we know from other research that these rather smaller nations are also strong in collaborating with other nations. Thus, it seems that smaller nations’ investments in research are especially effective in fostering (own) top-level research, if they are open to foreign research [53].
